# Intracardiac hydatid cyst located in right ventricular outflow tract: a rare site

**DOI:** 10.1007/s12055-021-01165-6

**Published:** 2021-04-02

**Authors:** Aviral Gupta, Sarvesh C. Mishra, Sushila Jaiswal, Shantanu Pande

**Affiliations:** 1 Department of Cardio-Vascular and Thoracic Surgery, Sanjay Gandhi Post-Graduate Institute of Medical Sciences, Lucknow, Uttar Pradesh India; 2 Department of Radiodiagnosis, Sanjay Gandhi Post-Graduate Institute of Medical Sciences, Lucknow, Uttar Pradesh India; 3 Department of Pathology, Sanjay Gandhi Post-Graduate Institute of Medical Sciences, Lucknow, Uttar Pradesh India

**Keywords:** Hydatid cyst, Intra-cardiac, Right ventricular outflow tract

## Abstract

Intracardiac hydatid cyst is relatively uncommon and involvement of right ventricular outflow tract is extremely rare. We report a rare case of intracardiac hydatid cyst involving the right ventricular outflow tract and do a review of literature.

## Introduction

Hydatid disease is a zoonotic parasitic infection caused by *Echinococcus granulosus*, *E. multilocularis*, or *E. vogeli*. Dogs and cats are definitive hosts of this parasite. Humans get infected as an accidental intermediate host, when they eat unwashed or uncooked vegetables or swallow the parasite eggs. Embryo of the parasite enters into blood circulation from intestine and can involve any organ. This infection commonly involves liver through portal vein, but if embryos bypass the liver, they reach the lungs via the inferior vena cava. They can also involve other organs like the heart. The frequency of cardiac involvement is less than 2% [1]. Intracardiac tumors, congenital cysts, and aneurysms form differential diagnosis of this lesion [2]. The left ventricle is the most common site of cardiac involvement [1]. The diagnosis of cardiac hydatid disease requires a combination of clinical suspicion, serologic tests, and cardiac imaging. Echocardiography is highly sensitive and specific in diagnosis of hydatid cysts and positive serological tests can help to diagnose this disease. Here, we present a rare case of intracardiac hydatid cyst involving right ventricular outflow tract (RVOT) and give a brief review of similar cases reported in the literature.

## Case report

**Fig. 1:**
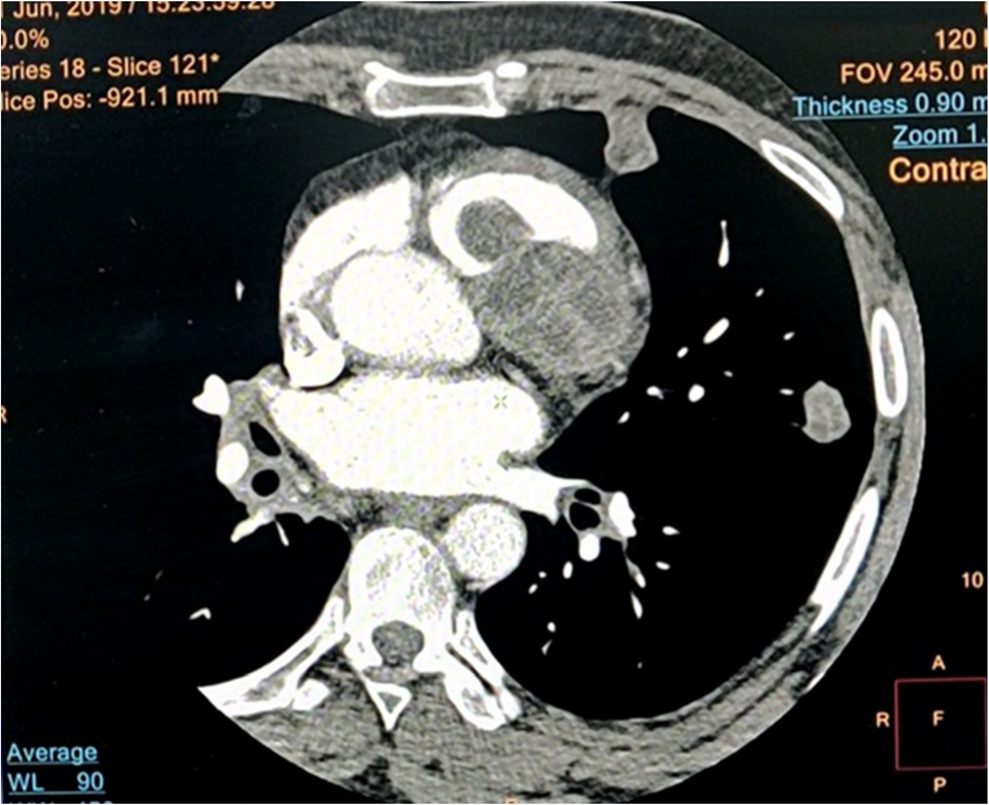
CT of the thorax showing multiloculated cystic lesion in the right ventricular outflow tract and multiple lung lesions

**Fig. 2:**
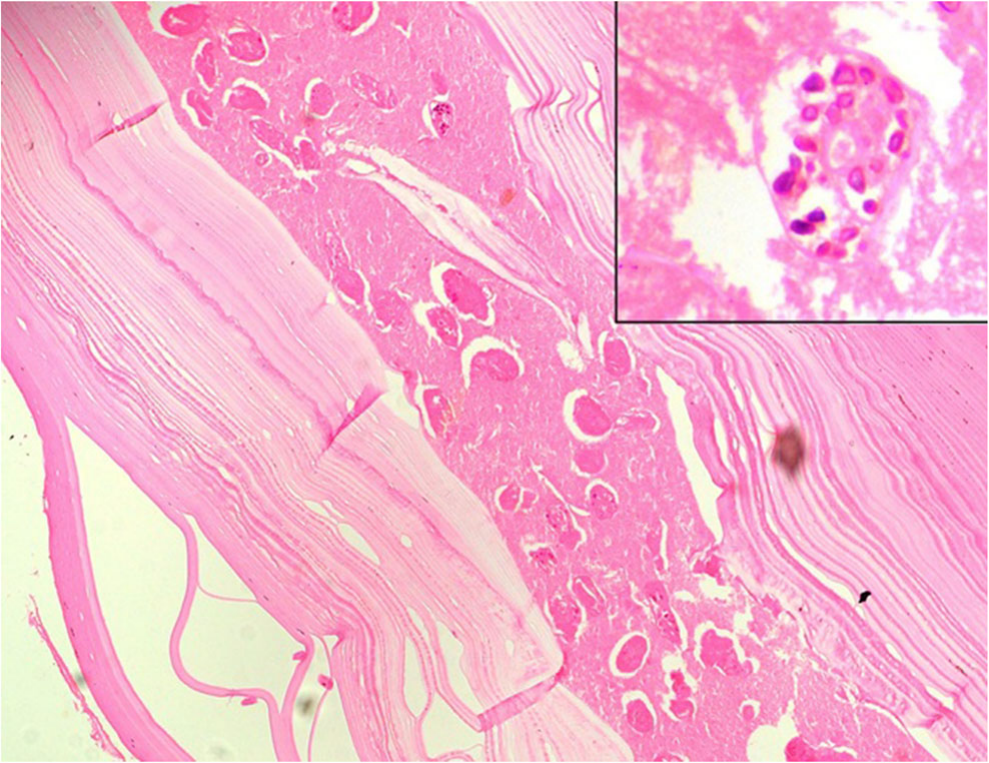
Acellular lamellated membranes of hydatid cyst (H&E, × 100) containing partially autolyzed brood capsules (inset, H&E, × 400)

A 46-year-old average built male, with no comorbidities, presented with occasional episodes of sudden hemoptysis over the last 4 years. During this period, he had intermittent cough with sputum, foul breath, and grade II dyspnea on exertion. He was initially seen by a local physician where he was suspected to have pulmonary tuberculosis. He received anti-tubercular medications for 6 months but the symptoms were not relieved. Thereafter, he was referred to our institute. Computerized tomography (CT) of the thorax was done which showed a multiloculated cystic lesion in relation to the RVOT and multiple lung lesions suggestive of hydatid disease (Fig. 1). Excision and marsupialization of the intracardiac cyst were performed and sent for histopathological examination. Histopathology showed the presence of acellular lamellated membranes of hydatid cyst with partially autolyzed brood capsules confirming the hydatid cyst (Fig. 2). The patient was discharged in stable condition after a few days and was prescribed oral albendazole 400 mg twice daily for 3 months. During close follow-up for 7 months, the patient was completely asymptomatic and did not have any episode of hemoptysis during or after surgery.

## Discussion

The left ventricle is the most frequent site of intracardiac hydatid (55 to 60%) [3]. Involvement of the interventricular septum is reported in 5 to 9% of cases [4]. Hydatid cysts originating from right atrial wall (3 to 4%) and right ventricular myocardium (15%) have also been reported. Involvement frequency of left atrial, pulmonary arterial, and pericardial localizations are reported to be 8%, 7 to 8%, and 5%, respectively. In the present case, hydatid cyst was located in the RVOT, which is an extremely rare site. Contractions of the heart provide a natural resistance to the presence of viable hydatid cyst, but this mechanism is not effective in all cases and the parasite can invade myocardial tissue in rare cases. Initially, the cyst grows slowly between the cardiac fibers and causes no signs or symptoms. Later, it may cause pericardial pain and dyspnea, invade the surrounding structure, obstruct the blood flow, and also invade the conductive system of heart and cause cardiac arrhythmias or conduction blocks [2, 5]. Some cases can mimic acute coronary syndrome [3], and may mandate coronary angiography (CAG). Echocardiography is sensitive for diagnosis of cardiac hydatid cyst. However, it is necessary to do CT scan or magnetic resonance imaging (MRI) to find additional information on the accurate location of lesion and relation of it with other structures. Most important major complication is the rupture of the cyst, which can trigger an anaphylactic shock or tamponade, systemic or pulmonary embolization, and compression of coronary branches. Cardiac surgery is the treatment of choice for most cases of cardiac hydatid cyst; however, the technique of surgery can be different. Scolicidal solutions such as iodine, ethanol, methylene blue, or hypertonic saline can be used to reduce the risk of leakage of fluid from cyst during cardiac surgery. After a successful surgical treatment, the duration of antibiotic therapy is variable. Until now, only seven cases of intracardiac hydatid cyst involving RVOT have been reported in literature to the best of our knowledge. The composite clinical data of these cases have been summarized in Table 1.

**Table 1:** Composite clinical data of patients with intracardiac hydatid cyst in the right ventricular outflow tract [5–10]

Case	Age (years) and sex (male or female)	Presenting complaints	Other organs involved	Imaging modality used for diagnosis	Surgery/procedure done	Outcome
1	44, male	Chest pain, palpitation, and dyspnea on exertion	No	Echocardiography	CPB cystectomy–capitonnage	Uneventful
2	26, female	Dyspnea on exertion	No	Echocardiography	CPB cystectomy–capitonnage	Uneventful
3	12, male	Dyspnea on exertion and recurrent syncope	No	Echocardiography	Patient collapsed in ward and underwent CPB cystectomy-capitonnage in emergency	Patient died due to cyst rupture and pulmonary embolism
4	16, male	Dyspnea, cough with hemoptysis	Bilateral lungs involved	Echocardiography	CPB cystectomy–capitonnage	Uneventful
5	35, female	Severe acute respiratory distress over several hours	No	None. Intracardiac hydatid cyst in RVOT was revealed on post-mortem	No surgery done	Patient died before any medical intervention
6	17, female	Dyspnea on exertion, palpitation	No	Echocardiography	CPB cystectomy–capitonnage	Uneventful
7	35, male	Dyspnea on exertion, palpitation	No	Echocardiography	CPB cystectomy–capitonnage	Uneventful
8	46, male (present case)	Dyspnea on exertion, cough with hemoptysis	Multiple lesions in the left lung	Echocardiography	CPB cystectomy–capitonnage	Uneventful

*CPB* cardio-pulmonary bypass

## Conclusion

Intracardiac hydatid cyst can sometimes rupture spontaneously and cause potentially fatal anaphylactic shock; hence, surgical excision is treatment of choice. The above is a case of intracardiac hydatid cyst located in the right ventricular outflow tract, which is an extremely rare site; and hence, we find it interesting to report.

### Availability of data and material

Not applicable.

### Code availability

Not applicable.
